# Draft Genome Sequences of Viridans Streptococci Causing Bacterial Endophthalmitis in Humans

**DOI:** 10.1128/MRA.00835-21

**Published:** 2021-10-21

**Authors:** Mary E. Marquart, K. Taylor Hellmann, Mary A. Carr, K. Michael Lovell, D. Ashley Robinson

**Affiliations:** a Department of Microbiology and Immunology, University of Mississippi Medical Center, Jackson, Mississippi, USA; University of Maryland School of Medicine

## Abstract

The viridans streptococci are a group of bacteria that are commensals of the oral cavity and pharynx. These species tend to cause severe cases of bacterial endophthalmitis with poor prognoses but remain largely uncharacterized in this context. Here, we report the whole-genome sequences of 21 strains of viridans streptococci isolated from endophthalmitis in humans.

## ANNOUNCEMENT

Streptococcal species that colonize the human oropharyngeal region, commonly called the viridans streptococci, comprise multiple species that play important roles in oral health and disease. These species have emerged as significant causes of bacterial endophthalmitis in humans, leading to blindness and/or enucleation (1). However, little is known regarding their pathogenic mechanisms in ocular infections. We aimed to sequence the genomes of clinical endophthalmitis strains of viridans streptococci to aid the study of these organisms as causes of ocular infections. Seventeen isolates, designated by the letter “E” followed by a number (Table 1), were kindly provided by Regis P. Kowalski (Charles T. Campbell Ophthalmic Microbiology Laboratory, University of Pittsburgh, Pittsburgh, PA). Four isolates, designated by numbers with hyphens, were generously provided by Darlene Miller (Bascom Palmer Eye Institute, University of Miami Health System, Miami, FL). These strains were previously identified to the species level (2).

Each strain was received on blood agar. The isolated colonies were cultured in Todd Hewitt broth containing 0.5% yeast extract at 37°C and 5% CO_2_ prior to genomic DNA extraction and purification using the DNeasy UltraClean microbial kit (Qiagen, Germantown, MD). Library preparation and sequencing were performed by the Molecular and Genomics Core Facility, University of Mississippi Medical Center, Jackson, MS. Libraries (150-bp paired-end format) were prepared using the Nextera XT DNA library preparation kit (Illumina, San Diego, CA), and sequencing was conducted using the Illumina NextSeq 500 platform. The sequence reads were trimmed for quality using Trimmomatic version 0.36 (3) and assembled *de novo* using SPAdes version 3.11.1 (4). The depth of coverage for the genome assemblies ranged from 146× to 2,064× (Table 1). The sequences were annotated using the NCBI Prokaryotic Genome Annotation Pipeline (PGAP). Genome assembly lengths ranged from 1.8 to 2.3 Mbp (Table 1), as expected for viridans streptococci (5, 6). The assembly quality was variable, with the number of contigs ranging from 45 to 1,354 and the contig *N*_50_ values ranging from 76,560 to 442,692 bp (Table 1). The annotated assemblies for all 21 strains were deposited in the genome submission portal of the National Center for Biotechnology Information (NCBI), National Library of Medicine, National Institutes of Health.

### Data availability.

The GenBank, Sequence Read Archive (SRA), and BioProject accession numbers are listed in Table 1. The NCBI BioSample accession numbers are available as links in GenBank.

**TABLE 1:** Genome information for streptococcal endophthalmitis strains

Strain	Species	GenBank accession no.	SRA accession no.	BioProject accession no.	Genome coverage (×)	Genome size (bp)	No. of contigs	Contig *N*_50_ (bp)	% GC
E619	Streptococcus oralis	JAGTPY000000000	SRP334189	PRJNA723877	181	2,038,810	117	133,191	41.5
E628	S. oralis	JAHCMD000000000	SRP334182	PRJNA729452	937	2,039,974	120	262,361	42.0
E636	Streptococcus salivarius	JAHFVU000000000	SRP334217	PRJNA732651	303	2,176,614	218	76,560	41.0
E647	Streptococcus mitis	JAHWWD000000000	SRP334218	PRJNA748383	209	1,941,891	136	82,400	40.2
E649	S. oralis	JAHCME000000000	SRP334216	PRJNA729458	261	2,325,945	1,354	210,456	42.3
E651	S. mitis	JAHFZD000000000	SRP334228	PRJNA732655	321	1,947,544	50	220,341	41.0
E653	Streptococcus vestibularis	JAHHFR000000000	SRP334250	PRJNA732899	2,064	2,038,817	1,026	84,869	40.8
E664	S. oralis	JAHHFS000000000	SRP334240	PRJNA732900	157	2,028,266	227	279,115	42.0
E665	S. oralis	JAHZTX000000000	SRP334297	PRJNA726988	200	2,101,270	723	324,883	41.2
E669	Streptococcus parasanguinis	JAHHFT000000000	SRP334241	PRJNA732901	424	2,266,699	126	91,101	42.7
E681	Streptococcus mutans	JAHHFU000000000	SRP334249	PRJNA732902	1,223	2,003,248	107	193,826	39.3
E684	S. oralis	JAHMJX000000000	SRP334304	PRJNA735205	227	1,971,377	45	159,424	42.5
E689	S. mitis	JAHMJY000000000	SRP334303	PRJNA735211	162	1,894,874	68	78,763	41.4
E697	S. oralis	JAGXBX000000000	SRP334296	PRJNA726981	245	2,013,862	119	301,460	42.2
E707	S. oralis	JAHMJZ000000000	SRP334299	PRJNA735246	202	2,167,014	76	88,806	43.3
E728	S. oralis	JAHCMC000000000	SRP334288	PRJNA729432	1,024	1,904,690	97	269,052	41.5
E734	S. oralis	JAHMSA000000000	SRP334285	PRJNA735250	830	2,239,686	790	442,692	42.1
11-4097	Streptococcus sp.^*a*^	JAGSFX000000000	SRP334287	PRJNA721732	424	2,259,404	667	119,508	41.6
11-6117	Streptococcus constellatus	JAHWWC000000000	SRP334292	PRJNA748382	213	1,869,421	70	244,417	38.3
14-4065	Streptococcus gordonii	JAHZSL000000000	SRP334293	PRJNA728738	179	2,249,747	73	292,239	40.5
14-4881	S. mitis	JAHFZC000000000	SRP334290	PRJNA732646	146	2,037,084	383	161,933	41.7

a Closest match for 11-4097: S. mitis (67% match to the type genome used by NCBI).
